# Mitochondrial matrix pH acidifies during anoxia and is maintained by the F_1_F_o_‐ATPase in anoxia‐tolerant painted turtle cortical neurons

**DOI:** 10.1002/2211-5463.12612

**Published:** 2019-03-14

**Authors:** Peter John Hawrysh, Leslie Thomas Buck

**Affiliations:** ^1^ Department of Cell and Systems Biology University of Toronto Canada; ^2^ Department of Ecology and Evolutionary Biology University of Toronto Canada

**Keywords:** anoxia, channel arrest, *Chrysemys picta bellii*, F_1_F_o_‐ATPase, K^+^/H^+^ exchanger, mitochondria, SNARF‐1

## Abstract

The western painted turtle (*Chrysemys picta bellii*) can survive extended periods of anoxia via a series of mechanisms that serve to reduce its energetic needs. Central to these mechanisms is the response of mitochondria, which depolarize in response to anoxia in turtle pyramidal neurons due to an influx of K^+^. It is currently unknown how mitochondrial matrix pH is affected by this response and we hypothesized that matrix pH acidifies during anoxia due to increased K^+^/H^+^ exchanger activity. Inhibition of K^+^/H^+^ exchange via quinine led to a collapse of mitochondrial membrane potential (Ψ_m_) during oxygenated conditions in turtle cortical neurons, as indicated by rhodamine‐123 fluorescence, and this occurred twice as quickly during anoxia which indicates an elevation in K^+^ conductance. Mitochondrial matrix pH acidified during anoxia, as indicated by SNARF‐1 fluorescence imaged via confocal microscopy, and further acidification occurred during anoxia when the F_1_F_o_‐ATPase was inhibited with oligomycin‐A, indicating that ΔpH collapse is prevented during anoxic conditions. Collectively, these results indicate that the mitochondrial proton electrochemical gradient is actively preserved during anoxia to prevent a collapse of Ψ_m_ and ΔpH.

**Abbreviations**
aCSF: artificial turtle cerebrospinal fluid
ATP: adenosine triphosphate
DMSO: dimethyl sulfoxide
FCCP: Carbonyl cyanide‐4‐(trifluoromethoxy)phenylhydrazone
mK^+^_ATP_: mitochondrial ATP‐sensitive K^+^ channel
mPTP: mitochondrial permeability transition pore
ROI: region of interest
SNARF: Seminaphtharhodafluor
SUR: sulfonylurea receptor
Ψm: membrane potential

Mitochondria are multifunctional organelles that regulate many critical cellular processes, such as the generation of adenosine triphosphate (ATP), Ca^2+^ signaling, reactive oxygen species generation, apoptosis, heat production, and hormone signaling 1, 2. Their ability to synthesize ATP through oxygen metabolism is central to many of these functions and ATP synthesis is directly coupled to the electrochemical gradient that exists across the mitochondrial inner membrane 3. The gradient is the combination of the mitochondrial membrane potential (Ψ_m_) and the pH gradient (ΔpH) and is referred to as the proton‐motive force, which is maintained by proton pumping from the mitochondrial matrix via the electron transport chain. It is the regulation of Ψ_m_, which is approximately −160 mV 4, that serves other functions beyond just ATP synthesis: Depolarization of Ψ_m_ increases mitochondrial respiration rate 5, stimulates release of mitochondrial Ca^2+^ 6, and increases fatty acid oxidation 7, while maintenance of a high Ψ_m_ drives protein import 8 and collapse of Ψ_m_ induces apoptosis 9. However, due to its control via proton flux, changes in Ψ_m_ can be intimately connected to changes in mitochondrial matrix pH, which also regulates several critical components of mitochondrial function besides mitochondrial respiration and ATP synthesis via the ATP synthase. Acidification of matrix pH prevents apoptotic induction via the mitochondrial permeability transition pore and reduces ROS production in isolated respiring mitochondria 10, 11. The activity of several mitochondrial ion transporters is influenced by changes in ΔpH, such as the Ca^2+^‐H^+^ exchanger, K^+^‐H^+^ exchanger, Na^+^‐H^+^ exchanger, and P_i_‐H^+^ phosphate cotransporter, which imports phosphate used for ATP synthesis 2, 12, 13, 14, 15.

While ΔpH changes are negligible during the transition from state 2/4 to state 3 respiration (0.05–0.1 pH units) 16, it is susceptible to greater changes during pathological changes such as ischemia/hypoxia: ΔpH collapses after 30–40 min of hypoxia to cytosolic values in rabbit cardiac myocytes, which is a change of approximately 0.9 units 17. The purpose of this investigation is to improve our understanding of how mitochondrial matrix pH changes in an organism that can tolerate extended periods of hypoxia and anoxia. The western painted turtle (*Chrysemys picta bellii*) can withstand over 4 months of anoxia at 3 °C, and it is able to do so through a series of cellular processes that reduce metabolic demand across different organ systems 18, 19, 20. Considerable attention has been given to understanding how turtle neurons respond to anoxia, given the brain's susceptibility to anoxia‐mediated cell death in mammalian models, and the mitochondria have been a central focus in many of these studies. Within a minute of anoxic saline perfusion, turtle mitochondria from cortical brain sheets depolarize via K^+^ influx through activated mitochondrial ATP‐sensitive K^+^ (mK^+^_ATP_) channels and a new depolarized state is maintained through proton efflux via reversal of the ATP synthase 21, 22. This depolarization is critical for the release of mitochondrial Ca^2+^ into the cytosol and for sustaining elevations in intracellular Ca^2+^ ([Ca^2+^]_i_), which ultimately result in downregulation of glutamatergic receptors and K^+^ channels in a process termed ‘channel arrest’ 22, 23, 24.

While we are beginning to understand how Ψ_m_ is regulated during anoxia in turtle pyramidal neurons, it is currently unknown how mitochondrial matrix pH is affected. Given that there is a relationship between mitochondrial K^+^ permeability and matrix pH, as mitochondrial K^+^ efflux is primarily regulated by 1 : 1 H^+^ exchange via the K^+^/H^+^ exchanger 25, it is likely that elevated mK^+^_ATP_ channel activity and consequent H^+^ influx through the K^+^/H^+^ exchanger leads to mitochondrial uncoupling and matrix acidification. Therefore, the purpose of this study was to determine how matrix pH changes during anoxia and how mK^+^_ATP_ channels and ion pumps/exchangers impact matrix pH during anoxia. We began this study with three primary hypotheses: (a) matrix pH acidifies during anoxia due to increased K^+^/H^+^ exchange following mK^+^_ATP_ channel activation, (b) inhibition of K^+^/H^+^ exchange will break the anoxic K^+^ circuit and will result in Ψ_m_ collapse, and (c) ΔpH is maintained by the reversed activity of the ATP synthase. Once conducted, this study demonstrated that when pyramidal neurons were made anoxic, there was a reduction in mitochondrial pH due to increased K^+^/H^+^ exchanger activity following mK^+^_ATP_ channel activity and this was balanced by H^+^ efflux via the F_1_F_o_‐ATPase (Fig. 1).

**Figure 1:**
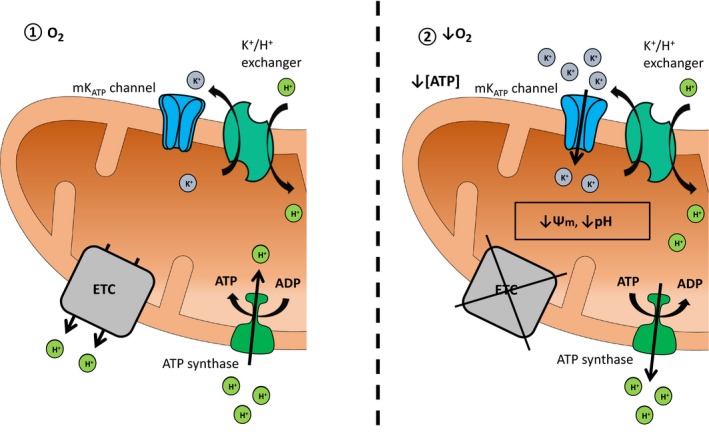
Summary of turtle mitochondrial activity during anoxia. During oxygenated conditions (1), the electron transport chain (ETC) maintains a proton gradient across the inner mitochondrial membrane that is used to produce ATP by shuttling protons back into the mitochondrial matrix through the ATP synthase. Mitochondrial ATP‐sensitive K+ (mK ^+^ _ATP_) channels remain in a closed state, and basal K+ homeostasis is maintained via the K^+^/H^+^ exchanger. Under anoxic conditions (2), mK ^+^ _ATP_ channels are activated by a local reduction in [ATP] and depolarize the mitochondrial membrane potential (Ψm). The elevation in K+ influx is balanced by K^+^/H^+^ exchange, which consequentially acidifies the mitochondrial matrix in the absence of ETC activity. To prevent complete collapse of the mitochondrial pH gradient, the ATP synthase hydrolyzes ATP to pump protons out of the matrix, maintaining a mildly depolarized and acidified matrix.

## Materials and methods

### Animal care and cortical slice preparation

This study was approved by the University of Toronto Animal Care Committee and conforms to the care and handling of animals as outlined in the Canadian Council on Animal Care's *Guide to the Care and Use of Experimental* Animals, Vol. 2. Adult turtles (*Chrysemys picta bellii*) were obtained from Niles Biological (Sacramento, CA, USA) and were housed in a large tank that was equipped with a freshwater flow‐through system at 18 °C. Turtles were kept on a constant 12 : 12 light cycle and were provided regular access to food, heat via a heat lamp, and a rock platform above the aquatic surface for basking.

Whole brains were rapidly excised from the cranium following decapitation and bathed in 3–5 °C artificial turtle cerebrospinal fluid (aCSF), which was comprised of (in mmol·L^−1^): 107 NaCl, 2.6 KCl, 1.2 CaCl_2_, 1.0 MgCl_2_, 2.0 NaH_2_PO_4_, 26.5 NaHCO_3_, 10.0 glucose, 5.0 imidazole, pH 7.4; osmolarity 285–290 mOsM. For SNARF‐1 experiments, whole brains were separated into hemispheres and cut into 300‐μm slices using a Vibratome 1000 plus sectioning system. Brain hemispheres were mounted on a steel block and embedded in 4% low‐melting point agarose (Type IX‐A, Sigma, Burlington, ON, Canada) on ice for a minimum of 5 min prior to slicing. For rhodamine‐123 experiments, cortical sheets were isolated from turtle brain and cut medially to produce a total of six cortical sheets and one cortical sheet was used per *N*‐value. No more than two cortical sheets were used per individual for any particular experimental group, such that a sample size of *N *=* *10 consisted of a minimum of five separate animals.

### Fluorometric assessment of mitochondrial matrix pH using SNARF‐1

Coronal slices were loaded with the cell‐permeable pH‐sensitive ratiometric dye 5‐(and‐6)‐carboxy SNARF‐1 Acetoxymethyl Ester (Life Technologies Inc., Burlington, ON, Canada), and mitochondria were labeled with MitoTracker Green FM (Life Technologies Inc.). Slices were incubated in 4 mL of ice‐cold aCSF containing 5 μmol·L^−1^ SNARF‐1 (from a 2 mmol·L^−1^ stock solution made from DMSO and 20% pluronic acid) for 2 h in an opaque container. Following this incubation period, slices were incubated in 200 nmol·L^−1^ MitoTracker Green for 60 min at approximately 22 °C. Dye‐loaded slices were housed in a flow‐through perfusion chamber over a #1.5 coverslip and held in place with a nylon‐strung slice anchor. Slices were imaged at 40× magnification using a TCS SP8 laser‐scanning confocal microscope (Leica Camera, Wetzlar, Germany). MitoTracker Green was excited using a 488‐nm laser and emission spectra were measured from 500 to 530 nm, while SNARF‐1 was excited using a 552‐nm laser and two separate channels were used to measure emissions from 565 to 605 nm and 610 to 700 nm due to the bimodal nature of its emission spectra. Fluorescence measurements were made at four separate points over the experimental period: Two measurements were taken at *t* = 0 and *t* = 10 min during oxygenated conditions, a third measurement after 15 min of anoxic and/or pharmacological treatments (*t* = 25), and a *t* = 35 measurement taken after a 10‐min recovery in oxygenated control aCSF. All light sources remained off between measurements to preserve the integrity of the fluorescent dyes, and dye‐loaded tissue was exposed to light for periods of up to approximately 1 min during fluorescence measurements. Fluorescence measurements were analyzed as the ratio of the measured emissions from 610–700 nm/565–605 nm and presented as the change in this ratio. Vertical stacks were taken over a span of 10–20 μm, and mitochondrial regions of interest (ROIs) were chosen based on co‐localization of both SNARF‐1 and MitoTracker dyes. ROIs were only chosen if they remained relatively stationary over the experimental time frame. A minimum of 10 ROIs were chosen per experiment, and the average change in fluorescence across these ROIs was used as one *N*‐value for statistical analysis.

### Fluorometric assessment of mitochondrial membrane potential using rhodamine‐123

Cortical sheets were loaded with the mitochondrial trans‐membrane potential‐sensitive dye rhodamine‐123 (Invitrogen, Burlington, ON, Canada) in an opaque vial containing 5 mL aCSF and 50 μmol·L^−1^ rhodamine‐123 [from a 25 mmol·L^−1^ rhodamine‐123 stock solution in dimethyl sulfoxide (DMSO)] for 50 min at 3–5 °C, followed by a 20‐min rinse in normal aCSF. Following dye loading, sheets were placed on a coverslip in a perfusion chamber system (RC‐26 open bath chamber with a P1 platform; Harvard Apparatus, Saint‐Laurent, QC, Canada). The chamber was gravity perfused from a 1‐L glass bottle attached to an intravenous dripper that contained aCSF gassed with 95% O_2_/5% CO_2_ to achieve oxygenated conditions. For experiments involving anoxia, a second 1‐L glass bottle with an attached intravenous dripper contained aCSF gassed with 95% N_2_/5% CO_2_ to achieve anoxic perfusion. Anoxic aCSF tubing was double jacketed, and the area between these jackets was gassed with 95% N_2_/5% CO_2_ to maintain anoxic conditions. A plastic cover with a hole for the electrode was placed over the saline bath, and the space between the cover and the bath surface was gently gassed with 95% N_2_/5% CO_2_ during anoxic conditions. For experiments involving pharmacological agent‐containing saline, this saline was bulk‐perfused using a second bottle that was bubbled with one of the aforementioned gas mixtures. Experiments were conducted at room temperature (22 °C).

Rhodamine‐123 was excited at 495 nm using a DeltaRamX highspeed random access monochromator and an LPS‐220B light source (Photon Technology International, London, ON, Canada) at a bandwidth of 4.5 nm using easyratiopro software (Photon Technology International). Light passed through a shutter for 1 s prior to every recording. Fluorescence emission measurements were acquired at 5‐s intervals using an Olympus BX51W1 microscope (Olympus Canada, Richmond Hill, ON, Canada) and Rolera‐MGi Digital EMCCD camera (QImaging, Surrey, BC, Canada). Baseline fluorescence was first measured for approximately 10 min to achieve a stable baseline, followed by treatment exposure for periods of 10–30 min. Drugs were applied by bulk perfusion as it was found that drug application using the fast‐step perfusion system occasionally produced artificial increases in fluorescence intensity because the force of the flow of the incoming saline moved the tissue slightly and shifted the area of focus. Following each treatment, oxygenated control aCSF was perfused onto the tissue to allow fluorescence to return to baseline. Changes in fluorescence were calculated as the difference between two parallel tangents obtained before and after treatment application, and this value was divided by baseline fluorescence to obtain a percent change in fluorescence. For each experimental recording, a minimum of 10 cells were randomly chosen and the average change in rhodamine‐123 fluorescence of these cells was used as one *N*‐value for statistical analysis. No observable changes in fluorescence occurred in response to anoxia in non‐dye‐loaded cells.

### Statistical analysis

Rhodamine‐123 and SNARF‐1 fluorescence data were analyzed using a one‐way ANOVA (Holm–Sidak method) following root arcsine transformation to compare the mean fluorescence between oxygenated controls and treatments. Data *N*‐values represent number of sheets/neurons analyzed per experimental data set, and no more than two cortical sheets were used from the same animal for any given experimental group.

## Results

### The increased flux through mitochondrial ATP‐sensitive potassium channels during anoxia is balanced by potassium/proton exchange

**Figure 2:**
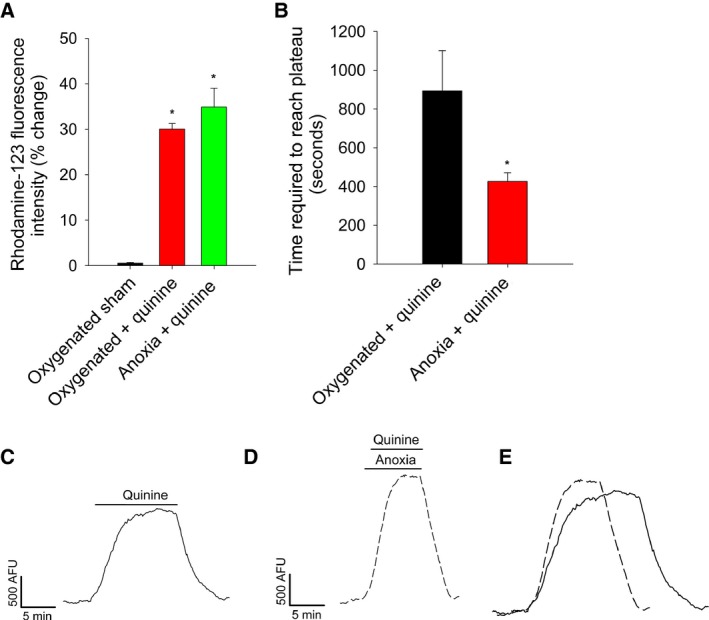
Mitochondrial membrane potential collapse in response to K^+^/H^+^ exchange inhibition with quinine occurs more rapidly during anoxia. A, percentage normalized changes in rhodamine‐123 fluorescence post treatment. Symbols indicate values significantly different from oxygenated controls (*) (*P* < 0.05), as indicated by a one‐way ANOVA (Holm–Sidak method). B, latency between the application of quinine to the fluorescence plateau during oxygenated conditions (*N *=* *8) or anoxia (*N *=* *9). Symbols indicate values significantly different from oxygenated controls (*) (*P* < 0.05), as indicated by a one‐way ANOVA (Holm–Sidak method). C‐E, sample data traces of rhodamine‐123 fluorescence from neurons treated with quinine during oxygenated conditions (C) or anoxia (D), which are superimposed in (E). Abbreviations: Arbitrary Fluorescence Units (AFU), minutes (min). All data are expressed as means ± SEM.

An increase in mK^+^_ATP_ channel activation plays a key role in the anoxic depolarization of pyramidal neuron mitochondria, which suggests that K^+^ influx also increases 22. Here, we investigated whether the deduced increase in K^+^ conductance and accumulation of matrix K^+^ would require an increase in K^+^ extrusion via the mitochondrial K^+^/H^+^ exchanger. Inhibition of the exchanger with quinine (1 mmol·L^−1^) resulted in collapse of Ψ_m_ both during oxygenated conditions and anoxia (30.1 ± 1.9, *N *=* *8, and 34.9 ± 4.1, *N *=* *9, respectively, Fig. 2A,C,D) but the collapse occurred significantly faster during anoxia (426.6 ± 44 s, *N *=* *9, *P* < 0.05; Fig. 2B,D,E) than during oxygenated conditions (893.2 ± 208.5 s, *N *=* *8, Fig. 2B,C,E). These data indicate that there was an increased mitochondrial K^+^ influx during anoxia and the K^+^/H^+^ exchanger balances this flux.

### The cell‐permeant pH sensitivity dye SNARF‐1 accumulates in mitochondria

**Figure 3:**
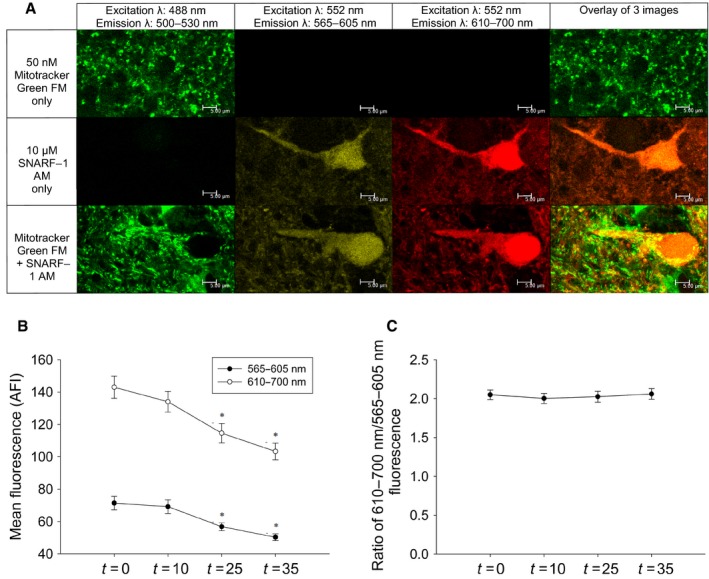
The pH‐sensitive dye SNARF‐1 maintains a constant fluorescence ratio and accumulates in mitochondria. A, Measurements of dye overlap in tissue loaded with 50 nmol·L^−1^ MitoTracker Green only (*N *=* *3), 10 μmol·L^−1^ SNARF‐1 only (*N *=* *3), or both 50 nmol·L^−1^ MitoTracker Green and 10 μmol·L^−1^ SNARF‐1. Analysis was performed on bright yellow regions from the image overlay indicating co‐localized uptake of both dyes. Scale bars represent 5 μm. B, mean SNARF‐1 fluorescence intensity of mitochondrial regions of interest (*N *=* *31) over the experimental period for two emission spectra (565–605 nm and 610–700 nm). Symbols (*) indicate data significantly different from recordings made at *t* = 0, as indicated by a one‐way ANOVA (Holm–Sidak method). C, normalized ratiometric (610–700 nm/565–605 nm) fluorescence of mitochondrially localized SNARF‐1 in response to oxygenated saline over the experimental period (*N *=* *31).

The cell‐permeant pH‐sensitive dye, SNARF‐1, was used to demonstrate how mitochondrial matrix pH changes in response to anoxic or pharmacological conditions. To confirm that mitochondrial uptake of SNARF‐1 occurred, tissue was incubated with SNARF‐1 and/or the mitochondrial‐specific label MitoTracker Green. Tissue incubated with MitoTracker alone emitted a measurable signal in the 500–530 nm range with no measurable signal appearing above 565 nm. Alternatively, tissue incubated with SNARF‐1 alone emitted measurable signals from 565 to 700 nm, with negligible overlap in the 500–530 nm range. For tissue incubated with both MitoTracker and SNARF‐1, regions exhibiting dye co‐localization were found surrounding the cell soma and dendritic extensions (Fig. 3A). During a 35 minutes recording period, SNARF‐1 fluorescence is significantly reduced after 25 minutes across both the 565–605 nm (*t* = 0: 71.3 ± 4.2; *t* = 10: 69.2 ± 4.3; *t* = 25: 56.8 ± 2.5; and *t* = 35: 50.3 ± 2.1, *N* = 31) and the 610‐700 nm spectra (*t* = 0: 143.0 ± 6.9; *t* = 10: 134.0 ± 6.3; *t* = 25: 114.6 ± 6.0; *t* = 35: 103.3 ± 5.1, *N* = 31, Fig. 3B). However, when these values are taken as a ratio over the same time period, there is no change in the ratio under control conditions (*t* = 0: 2.1 ± 0.1; *t* = 10: 2.0 ± 0.1; *t* = 25: 2.0 ± 0.1; *t* = 35: 2.1 ± 0.1; *N* = 31, Fig. 3C). This indicates that the ratio of SNARF‐1 fluorescence from 610–700 nm/565–605 nm is not susceptible to photo‐bleaching. Taken together, this demonstrates that SNARF‐1 is taken up by the cell and accumulates in mitochondria.

### Mitochondrial matrix pH is stable during oxygenated conditions but acidifies during anoxia, independently of potassium/proton exchange

**Figure 4:**
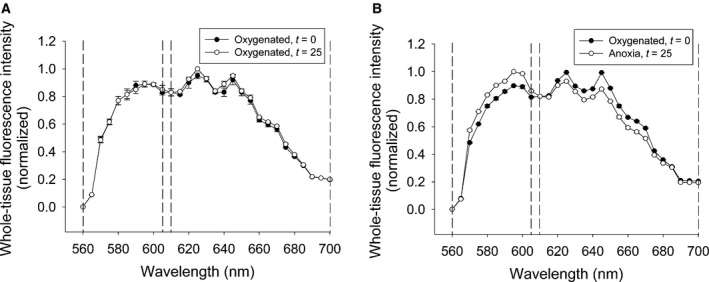
A leftward spectral shift of SNARF‐1 occurs in response to anoxia‐mediated acidification of turtle brain. Measured emission spectra across whole‐tissue ROIs of turtle brain during oxygenated controls at *t* = 0 and *t* = 25 min (A, *N *=* *5), or oxygenated conditions at *t* = 0 min followed by anoxia at *t* = 25 min (B, *N *=* *13). Regions bordered by dotted lines correspond to the 565–605 nm and 610–700 nm regions measured for ratiometric analysis, respectively.

During oxygenated conditions, there is a progressive reduction in SNARF‐1 fluorescence which likely occurs due to photo‐bleaching and/or dye extrusion. A significant reduction in fluorescence intensity with respect to *t* = 0 was measured at *t* = 25 and 35 min for the 565–605 nm and 610–700 nm spectra (Fig. 4B). However, when fluorescence was presented as a ratio of 610–700 nm/565–605 nm, there were no observable differences in this ratio (Fig. 4C) or shifts in the emission spectrum (Fig. 4A) over the experimental period.

**Figure 5:**
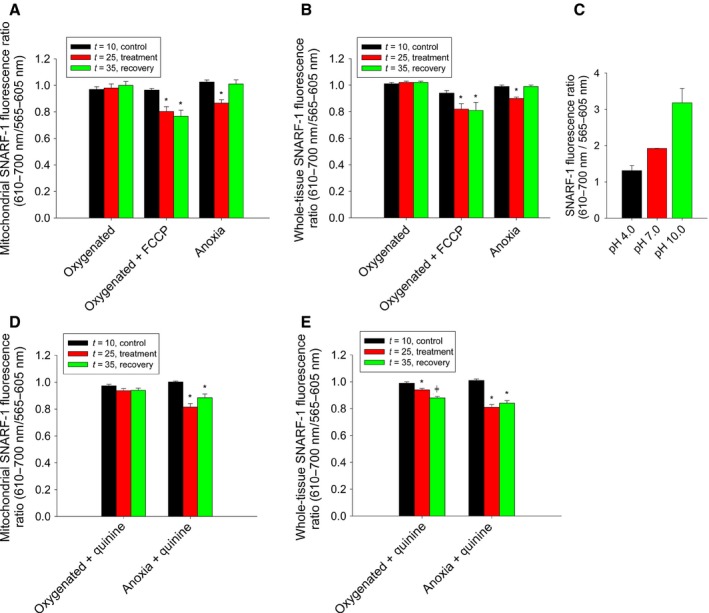
The mitochondrial matrix acidifies in response to anoxia and this occurs independently of K^+^/H^+^ exchange. A, normalized ratiometric (610–700 nm/565–605 nm) fluorescence of mitochondrially localized SNARF‐1 in response to oxygenated saline over the experimental period (*N *=* *5), the protonophore FCCP (*N *=* *4), or anoxic saline (*N* = 5). B, normalized ratiometric fluorescence of whole‐tissue SNARF‐1 as treated in A. C, fluorescence ratios of cell‐impermeant SNARF‐1 at pH 4.0 (*N* = 6), 7.0 (*N* = 3), and 10.0 (*N* = 4). D, normalized ratiometric fluorescence of mitochondrially localized SNARF‐1 in response to K^+^/H^+^ exchange inhibition via quinine during oxygenated or anoxic conditions. E, normalized ratiometric fluorescence of whole‐tissue SNARF‐1 as treated in B. An asterisk (*) indicates values significantly different (*P* < 0.05) from oxygenated controls (*t* = 10) taken within the same experimental group, as indicated by a one‐way ANOVA (Holm–Sidak method). Data were normalized to fluorescence values taken at *t* = 0. All data are expressed as means ± SEM.

Fluorescence ratios normalized to values obtained at *t* = 0 remained stable during oxygenated conditions at *t* = 10, 25, and 35 min for both mitochondrial ROIs (0.97 ± 0.02%, 0.98 ± 0.03%, and 1.00 ± 0.03%, respectively, *N *=* *5, Fig. 5A) and whole‐tissue ROIs (1.01 ± 0.01%, 1.02 ± 0.01%, and 1.02 ± 0.01%, respectively, *N *=* *5, Fig. 5B). However, there was a leftward shift in the emission spectra after exposure to 15 min of anoxia (Fig. 5B), which is indicative of dye acidification, and this was demonstrated by a significant reduction in the fluorescence ratio during anoxia and a recovery of values to pre‐anoxic levels following 10 min of re‐oxygenation, as seen in mitochondrial ROIs (*t* = 10: 1.03 ± 0.02%, *t* = 25: 0.87 ± 0.03%, *t* = 35: 1.01 ± 0.03%, *N *=* *5, *P* < 0.05, Fig. 5A) or whole‐tissue ROIs (*t* = 10: 0.99 ± 0.01%, *t* = 25: 0.90 ± 0.01%, *t* = 35: 0.99 ± 0.01%, *N *=* *5, *P* < 0.05, Fig. 5B). To demonstrate that this ratio reduction was due to dye acidification, tissue was exposed to the protonophore FCCP, which also resulted in a significant reduction in the fluorescence ratio but did not recover following washout in either mitochondrial ROIs (*t* = 10: 0.97 ± 0.01%, *t* = 25: 0.80 ± 0.04%, *t* = 35: 0.77 ± 0.05%, *N *=* *4, *P* < 0.05, Fig. 5A) or whole‐tissue ROIs (*t* = 10: 0.94 ± 0.02%, *t* = 25: 0.82 ± 0.04%, *t* = 35: 0.81 ± 0.06%, *N *=* *4, *P* < 0.05, Fig. 5B). Furthermore, acidification of the cell‐impermeant form of SNARF‐1 resulted in a reduction of the fluorescence ratio as the pH was reduced from pH = 10 (3.18 ± 0.40, *N *=* *4, Fig. 5C) to pH = 7 (1.92 ± 0.01, *N *=* *4, Fig. 5C) and pH = 4 (1.31 ± 0.14, *N *=* *6, Fig. 5C). Taken together, these data suggest that a reduction in the fluorescence ratio of SNARF‐1 indicates a lowered pH and the mitochondrial matrix acidifies in response to anoxia.

To determine whether the K^+^/H^+^ exchanger is a key regulator of mitochondrial matrix pH during anoxia, 1 mmol·L^−1^ of quinine was applied. We hypothesized that inhibition of K^+^/H^+^ exchange during anoxia would prevent the elevated influx of H^+^ that would occur in response to K^+^ efflux and would thus mitigate mitochondrial acidification. During oxygenated conditions, quinine had no observable effect on mitochondrial pH (*t* = 10: 0.97 ± 0.01%, *t* = 25: 0.94 ± 0.01%, *t* = 35: 0.94 ± 0.02%, *N *=* *4, Fig. 5D) but resulted in a reduction in the whole‐tissue fluorescence ratio (*t* = 10: 0.99 ± 0.01%, *t* = 25: 0.94 ± 0.01%. *t* = 35: 0.88 ± 0.01%, *N *=* *4, *P* < 0.05, Fig. 5E). However, during anoxia and quinine application, the anoxia‐mediated reduction in mitochondrial matrix pH was still observed but did not recover following aCSF washout (*t* = 10: 1.00 ± 0.01%, *t* = 25: 0.82 ± 0.03%, *t* = 35: 0.89 ± 0.03%, *N *=* *4, *P* < 0.05, Fig. 5D) with the same effect being observed in whole‐tissue ROIs (*t* = 10: 1.01 ± 0.01%, *t* = 25: 0.81 ± 0.02%, *t* = 35: 0.84 ± 0.02%, *N *=* *4, *P* < 0.05, Fig. 5E). From these data, we conclude that while K^+^/H^+^ exchange occurs during anoxia, it is not necessary for mitochondrial acidification to occur.

### Mitochondrial matrix pH is maintained via F_1_F_o_‐ATPase activity during anoxia

**Figure 6:**
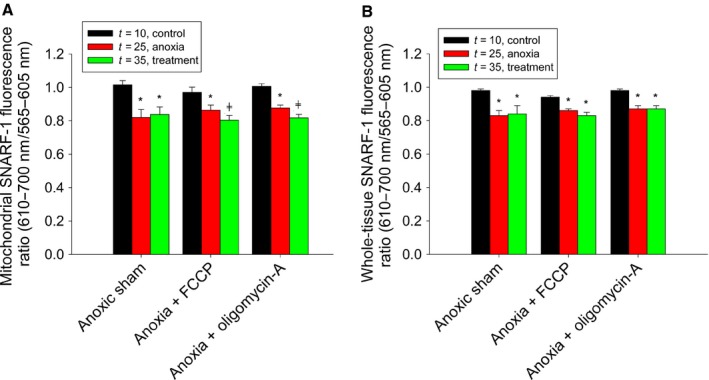
Mitochondrial matrix pH is prevented from collapsing by the ATP synthase during anoxia. A, normalized ratiometric (610–700 nm/565–605 nm) fluorescence of mitochondrially localized SNARF‐1 in response to exposure to anoxic saline (*N* = 4) the protonophore FCCP (*N* = 6) or the ATP synthase inhibitor oligomycin‐A (*N* = 5) during anoxic conditions. B, normalized ratiometric fluorescence of whole‐tissue SNARF‐1 as treated in A. Symbols indicate data significantly different (*P* < 0.05) from oxygenated controls taken at *t* = 10 (*) or anoxic controls taken at *t* = 25 (╪) within the same experimental group, as indicated by a one‐way ANOVA (Holm–Sidak method). Data were normalized to fluorescence values taken at *t* = 0. All data are expressed as means ± SEM.

Mitochondrial pH measurements taken during anoxia were extended from *t* = 25 to *t* = 35, and the anoxic reduction in the fluorescence ratio was found to be stable across this time period in both mitochondrial ROIs (*t* = 10: 1.02 ± 0.03%, *t* = 25: 0.82 ± 0.05%, *t* = 35: 0.84 ± 0.05%, *N *=* *4, *P* < 0.05, Fig. 6A) and whole‐tissue ROIs (*t* = 10: 0.98 ± 0.01%, *t* = 25: 0.83 ± 0.03%, *t* = 35: 0.82 ± 0.03%, *N *=* *4, *P* < 0.05, Fig. 6B). To determine whether mitochondrial matrix pH is regulated similar to Ψ_m_, we applied 20 μmol·L^−1^ of the protonophore FCCP following the measurement taken at *t* = 25. The reduction in the SNARF‐1 fluorescence ratio during anoxia was further reduced following treatment with FCCP in mitochondrial ROIs (*t* = 10: 0.97 ± 0.03%, *t* = 25: 0.86 ± 0.03%, *t* = 35: 0.80 ± 0.03%, *N *=* *6, *P* < 0.05, Fig. 5A) but not whole‐tissue ROIs (*t* = 10: 0.94 ± 0.01%, *t* = 25: 0.86 ± 0.01%, *t* = 35: 0.83 ± 0.02%, *N *=* *6, *P* < 0.05, Fig. 6B), suggesting that mitochondrial matrix pH is regulated during anoxic conditions. To demonstrate that the F_1_F_o_‐ATPase maintains ΔpH by pumping protons out of the matrix, 10 μmol·L^−1^ oligomycin‐A was used during extended anoxic conditions. When applied following the *t* = 25 measurement, oligomycin‐A resulted in a further reduction in the fluorescence ratio in mitochondrial ROIs (*t* = 10: 1.00 ± 0.02%, *t* = 25: 0.88 ± 0.02%, *t* = 35: 0.82 ± 0.02%, *N *=* *6, *P* < 0.05, Fig. 6A) but not whole‐tissue ROIs (*t* = 10: 0.98 ± 0.01%, *t* = 25: 0.87 ± 0.02%, *t* = 35: 0.87 ± 0.02%, *N *=* *6, *P* < 0.05, Fig. 6B), which demonstrates that oligomycin‐A acts specifically at the mitochondrial level. Taken together, these data suggest that Ψ_m_ is protected during anoxic conditions by a reversal of the proton flux via the F_1_F_o_‐ATPase, which is illustrated here by maintenance of mitochondrial matrix pH.

## Discussion

In this study, we demonstrated that when pyramidal neurons were made anoxic, there was a reduction in mitochondrial pH that was regulated by H^+^ efflux via the F_1_F_o_‐ATPase. The increase in K^+^ efflux mediated by the K^+^/H^+^ exchanger in response to elevated mK^+^_ATP_ channel activity 21 is balanced by an increase in H^+^ influx, which contributes to the acidification of matrix pH (Fig. 1). This investigation corroborates our previous work which demonstrated that the anoxia‐mediated depolarization of Ψ_m_ via mK^+^_ATP_ channel activation is balanced by H^+^ efflux via the F_1_F_o_‐ATPase and Ψ_m_ is maintained at a depolarized state 22. While there are no commercially available mitochondrial K^+^‐sensitive dyes that could be used to measure mitochondrial K^+^ flux, we were able to utilize quinine and rhodamine‐123 fluorescence to deduce mitochondrial K^+^ flux. The importance of this current work rests in the demonstration that ΔpH does not fully collapse during anoxic conditions: However, application of the F_1_F_o_‐ATPase inhibitor oligomycin‐A during anoxia caused further matrix acidification. There is concern regarding the use of quinine as a K^+^/H^+^ exchanger inhibitor, given that it is also an inhibitor of mK^+^_ATP_ channels by acting on the sulfonylurea receptor (SUR) component 26. However, the structure of mK^+^_ATP_ channels in turtle neurons remains debatable. Typically, plasmalemmal K^+^_ATP_ channels are octamers that consist of a combination of four inwardly rectifying potassium channel (Kir) subunits (either Kir6.1 or Kir6.2) and four SUR subunits (SUR1, SUR2A, and SUR2B), but concrete evidence supporting similar structures in mitochondria has been controversial. Mitochondrial K^+^_ATP_ channel activity has been reported to remain intact in the absence of several of these subunits in different model organisms, and immunogold electron microscopy has revealed the presence of Kir6.1 and Kir6.2 subunits in the inner mitochondrial membrane of rat cardiomyocytes but not SUR1 or SUR2 subunits, although a truncated SUR2 subunit was localized to mouse heart mitochondria 27, 28. Therefore, it is possible that such a system is also present in the turtle given that a pharmacological response to quinine still occurs (Fig. 2). It is also worth acknowledging that SNARF‐1 fluorescence did not recover following quinine application during anoxia and that, while resistant to anoxic episodes, turtle mitochondria may not be able to tolerate pharmacological insults that disrupt Ψ_m_ regulation during anoxia and may need a longer time frame to recover than what was utilized here.

It should be noted that these experiments were conducted at a temperature (22 °C) that would not normally be associated with anoxic overwintering in nature, and as such, the response we observed may differ in nature due to the temperature sensitivity of the response. While the degree of anoxia‐mediated metabolic depression is similar between 25 °C and 10 °C in isolated hepatocytes 29, there is a 30‐fold difference in whole‐animal anaerobic metabolism from 20 °C to 3 °C 30 which indicates that each organ system may possess a unique sensitivity to temperature. This is further demonstrated by brain Na^+^/K^+^ ATPase pumps, which undergo a 50% reduction in pump density during anoxia from 21 °C to 5 °C 31. Mitochondrial K^+^/H^+^ antiporters possess a 66% reduction in activity from 21 °C to 4 °C in bovine heart mitochondria 32, which may act similarly in turtle brain. Additionally, pH decreases with lowered temperature in anoxic turtle plasma 30 and cytosol 33 and mitochondrial ΔpH increased with decreased temperature in hypoxia‐tolerant carp red muscle 34. These results suggest that the observations made in this investigation could be more pronounced under natural conditions.

The prevention of collapse in mitochondrial ΔpH is a noteworthy characteristic of turtle mitochondria, given that anoxia‐mediated Ca^2+^ release occurs through a cyclosporine‐A‐sensitive mechanism proposed to be mitochondrial permeability transition pore (mPTP) opening 22. Opening of the mPTP leads to a dramatic decline of ΔpH, as indicated in cultured rat cortical neurons: glutamate‐induced elevations in Ca^2+^ lead to a reduction in ΔpH across the mitochondrial membrane from 0.8 to 0.3 units and this can be prevented with Sr^2+^, an mPTP inhibitor 35. Given that mitochondria have a low inherent capacity for H^+^ buffering 36, it is likely that the turtle has adapted to actively regulate ΔpH, as it does Ψ_m_, to avoid being confronted by the consequences of its destabilization. Mitochondrial ΔpH is largely unchanged following 30 min of anoxia in rat hepatocytes 4, suggesting that it may be beneficial for the turtle to reduce matrix pH since ΔpH does not change in anoxia‐intolerant organisms. IF_1_, an endogenous inhibitory protein of the mitochondrial F_1_F_o_‐ATPase, possesses a histidine‐rich segment between residues 48 and 70 that is implicated in pH‐dependent conformational changes which affect protein activity 37. The ATPase inhibitory activity of IF_1_ increases as matrix pH decreases from 8.0 to 6.7, due to a change in its oligomerization state from inactive tetramers to active dimers 38, 39. The modest acidification of matrix pH may contribute to its ATPase inhibitory activity during anoxia: F_1_F_o_‐ATPase activity is reduced by 80% and 85% in anoxic turtle brain and heart, respectively, without a change in protein content which suggests that activity is structurally regulated 40, 41. Acidification of matrix pH could therefore play a role in ATP conservation in the turtle and prevent excessive ATP hydrolysis via the F_1_F_o_‐ATPase. Matrix pH is also an essential factor which determines ROS production, as a decrease in matrix pH in respiring mitochondria in the presence of phosphate results in a steady decrease in ROS generation due to semiquinone radical protonation and reduced superoxide production 11. It may therefore be one of the contributing factors that prevents a surge of ROS following re‐oxygenation, which is not seen in turtle neurons 42.

## Conflict of interest

The authors declare no conflict of interest.

## Author contributions

PJH and LTB made equal contributions to conceiving the study. PJH designed the experiments, performed all experimental work, analyzed all experimental data, and wrote the first draft of the manuscript. LTB edited and drafted the final manuscript. All authors gave final approval for publication.
